# Neues zur Diagnostik und Therapie der Herzinsuffizienz

**DOI:** 10.1007/s00059-021-05062-x

**Published:** 2021-08-31

**Authors:** Jan Wintrich, Ann-Kathrin Berger, Yvonne Bewarder, Insa Emrich, Jonathan Slawik, Michael Böhm

**Affiliations:** Klinik für Innere Medizin III – Kardiologie, Angiologie und Internistische Intensivmedizin, Universitätsklinikum des Saarlandes, Kirrbergerstraße, 666421 Homburg/Saar, Deutschland

**Keywords:** Herzinsuffizienz, Kardiale Dekompensation, LCZ696, SGLT2-Inhibitoren, Vericiguat, Heart failure, Cardiac decompensation, LCZ696, Sodium-glucose transporter 2 inhibitors, Vericiguat

## Abstract

Inzidenz und Prävalenz der Herzinsuffizienz steigen weltweit. Trotz zahlreicher wissenschaftlicher und klinischer Innovationen ist sie weiterhin mit einer hohen Morbidität und Mortalität behaftet, sodass eine leitliniengerechte Diagnostik und Therapie von entscheidender Bedeutung sind. Die kardiale Dekompensation zählt zu den häufigsten Aufnahmegründen in deutschen Krankenhäusern. Somit stellt die Behandlung herzinsuffizienter Patienten eine erhebliche Herausforderung für das deutsche Gesundheitssystem dar. Dieser Artikel fasst die neuesten wissenschaftlichen Erkenntnisse zur akuten und chronischen Herzinsuffizienz der Jahre 2018 bis 2020 zusammen.

## Abstract (en)

The incidence and prevalence of heart failure are increasing worldwide. Despite numerous scientific and clinical innovations the mortality and morbidity rates in heart failure patients remain high, so that guideline-conform diagnostics and treatment are of decisive importance. Cardiac decompensation is one of the leading reasons for hospital admissions in Germany. Thus, the treatment of patients with heart failure represents a substantial challenge for the German healthcare system. This article highlights the latest scientific knowledge on acute and chronic heart failure from the years 2018–2020.

Diese Arbeit stellt die wesentlichen wissenschaftlichen Erkenntnisse und Studienergebnisse aus den Jahren 2018 bis 2020 zur akuten und chronischen Herzinsuffizienz zusammen. Aufbauend auf den aktuellen Leitlinien der Europäischen Gesellschaft für Kardiologie (ESC; [1]) werden innovative Strategien im Bereich der Herzinsuffizienzdiagnostik und -therapie erläutert. Des Weiteren erfolgt die Vorstellung noch nicht abgeschlossener bzw. geplanter Studien, die in unmittelbarer Zukunft einen entscheidenden Einfluss auf die klinische Praxis nehmen könnten.

## Neue diagnostische Strategien

**Figure 1:**
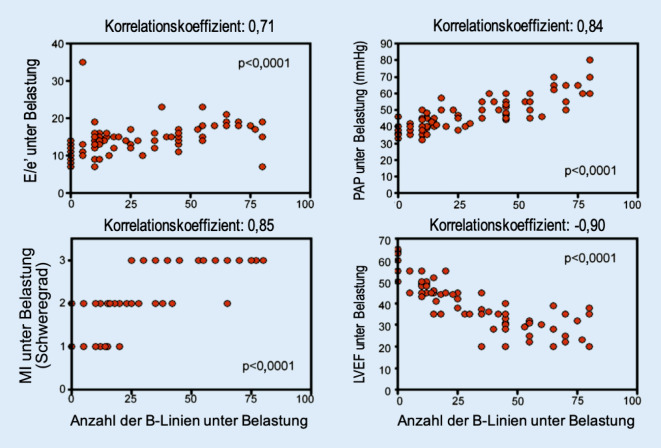

Der schnelle und einfache Nachweis einer kardialen Dekompensation kann in Notfallsituationen von großer Bedeutung sein, um schwerwiegende Komplikationen, z. B. ein beatmungspflichtiges Lungenödem, zu vermeiden. In den letzten Jahren konnte hierbei die Beurteilung sog. B‑Linien mittels der thorakalen Ultraschalldiagnostik an Bedeutung gewinnen. Die B‑Linien entsprechen Schallschatten an interlobulären Septen und kennzeichnen einen erhöhten Wassergehalt der Lungen [2]. Bereits in frühen notfallmedizinischen Studien wurde gezeigt, dass der sonographische Nachweis von B‑Linien mit der BNP(„brain natriuretic peptide“)-Konzentration und radiologischen Dekompensationszeichen assoziiert ist [2]. Darüber hinaus kann die thorakale Ultraschalldiagnostik in Kombination mit einer Stressechokardiographie zur Einschätzung hämodynamischer Parameter und der Prognose genutzt werden, wie eine Studie an 103 Herzinsuffizienzpatienten im NYHA(New York Heart Association)-Stadium I–III zeigen konnte. Während des Stressechos ließ sich bei diesen Patienten ein signifikanter Anstieg der B‑Linien dokumentieren, welcher positiv mit der Höhe des belastungsinduzierten pulmonalarteriellen Drucks und der Schwere der Mitralinsuffizienz unter Belastung sowie negativ mit der linksventrikulären Ejektionsfraktion (LVEF) korrelierte ([3]; Abb. 1). Ferner ging der Nachweis von mindestens 30 B-Linien unter körperlicher Belastung mit einem höheren kardiovaskulären Risiko (kombinierter Endpunkt aus Herzinsuffizienzhospitalisierung, Myokardinfarkt und Gesamtsterblichkeit) einher. Aus einer weiteren Untersuchung an 162 hospitalisierten Herzinsuffizienzpatienten geht hervor, dass anhand der Anzahl der B‑Linien bei Entlassung das Risiko einer Herzinsuffizienzrehospitalisierung und das Mortalitätsrisiko abgeschätzt werden kann [4].

**Figure 2:**
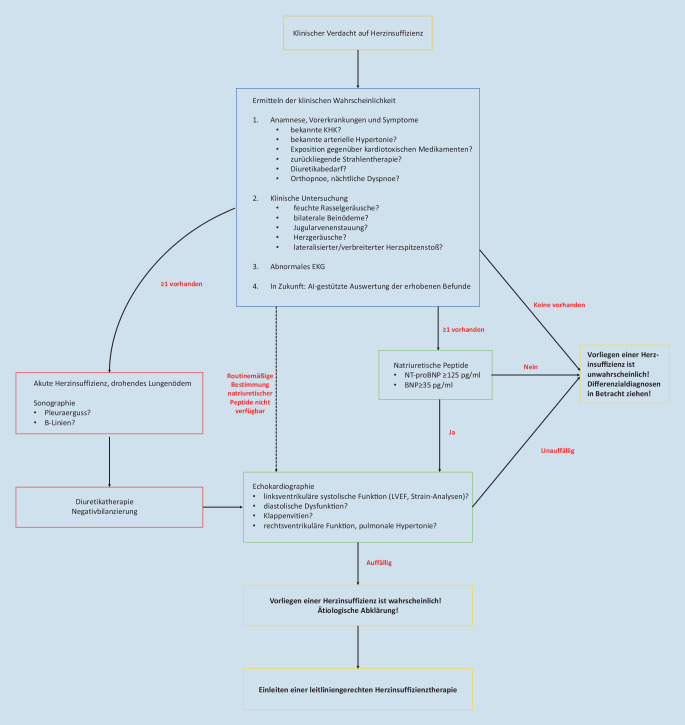

Bei der Durchführung einer thorakalen Ultraschalluntersuchung ist ein einheitliches Vorgehen notwendig, um reproduzierbare und vergleichbare Messwerte zu erhalten. Daher wurde in einem Konsensusdokument der Heart Failure Association (HFA) der ESC ein standardisiertes Schema beschrieben, welches zur Abklärung einer kardialen Dekompensation angewandt werden sollte [5]. In Zukunft könnte der Stellenwert der thorakalen Ultraschalldiagnostik im Bereich der akuten und chronischen Herzinsuffizienz weiter steigen, da sie schnell und leicht zugänglich durchgeführt werden kann (Abb. 2).

Die frühzeitige Diagnose einer Herzinsuffizienz ist essenziell, um eine leitliniengerechte Therapie rechtzeitig einzuleiten und damit die Prognose der Patienten zu verbessern. In Regionen, in denen nur eine geringe Anzahl kardiologischer Abteilungen bzw. Praxen vorzufinden ist, kann die Diagnose der Herzinsuffizienz jedoch erschwert sein. In Zukunft könnte hierbei die Anwendung von Künstlicher Intelligenz („artificial intelligence“, AI) einen entscheidenden Vorteil bedeuten. Wie eine südkoreanische Studie zeigen konnte, lässt sich eine Herzinsuffizienz mit Hilfe von AI sicher erkennen. Hierzu wurde mithilfe retrospektiver Daten (u. a. Alter, Geschlecht, Symptomatik, Laborwerte, echokardiographische Parameter) von 1198 Patienten ein Diagnosealgorithmus erarbeitet und validiert [6]. Im Anschluss erfolgte der prospektive Vergleich einer AI-basierten Herzinsuffizienzdiagnose gegenüber einer Referenzdiagnose durch Herzinsuffizienzexperten an 97 Patienten, die mit Dyspnoe ambulant vorstellig wurden. Bei 44 % der Patienten konnte eine Herzinsuffizienz nachgewiesen werden. In 98 % der Fälle entsprach die AI-basierte Diagnose der Referenz der Herzinsuffizienzexperten [6]. Des Weiteren erlauben AI-gestützte Auswertungen echokardiographischer Bilder eine sehr genaue Ermittlung der LVEF und können zur Minimierung der Interobservervariabilität beitragen [7]. Darüber hinaus ist es durch die Anwendung von AI-gestützten Verfahren möglich, Patienten mit einer eingeschränkten LVEF von weniger als 35 % durch das Schreiben eines 12-Kanal-EKGs mit einer Sensitivität von 74 % und einer Spezifität von 87 % zu identifizieren [8]. Hierbei handelt es sich aber um Ergebnisse einer retrospektiven Studie. Die prospektive, randomisierte EAGLE-Studie [9] wird untersuchen, ob die AI-gestützte EKG-Diagnostik als ein Screeningverfahren zum Erkennen einer Herzinsuffizienz in der klinischen Routine in Frage kommt (NCT04000087).

## Akute Herzinsuffizienz

### Versorgungsaspekte

Eine kardiale Dekompensation, die eine stationäre Behandlung notwendig macht, geht mit einer ungünstigen Prognose einher. Daher sind eine adäquate Versorgung im Krankenhaus sowie ein schnelles Einleiten therapeutischer Maßnahmen essenziell. Einen entscheidenden Faktor stellt hierbei die Dichte versorgender Einrichtungen (Krankenhäuser, Arztpraxen) dar. Sowohl in den neuen Bundesländern als insbesondere auch im Südosten und Nordosten Deutschlands sowie im Saarland und in Rheinland-Pfalz besteht eine geringe Dichte versorgender Einrichtungen. Eine Auswertung der Versicherungsdaten von etwa 87 % aller deutschen Einwohner zeigt jedoch, dass in diesen Regionen eine hohe Prävalenz herzinsuffizienter Patienten vorliegt [10]. Zusätzlich ist das Risiko, an einer Herzinsuffizienz zu erkranken, in ländlichen Gebieten mit einer geringen Einwohnerzahl gegenüber großen, urbanen Zentren um 40 % erhöht. Um eine optimale Versorgung herzinsuffizienter Patienten innerhalb Deutschlands zu bewerkstelligen, sollten diese Aspekte bei der Planung zukünftiger Projekte und Einrichtungen berücksichtig werden.

### Therapie der akuten Herzinsuffizienz

Obwohl die akute Herzinsuffizienz mit einer hohen Letalität verbunden ist, sind evidenzbasierte Therapien, die mit einer Reduktion kardiovaskulärer Endpunkte einhergehen, aktuell nicht verfügbar. Die Behandlung der akuten Herzinsuffizienz beschränkt sich in aller Regel auf die Einleitung rekompensierender Maßnahmen, allen voran einer diuretischen Therapie, wodurch die Symptomatik der Patienten gelindert werden kann. Die Applikation der Diuretika kann hierbei auf verschiedene Arten erfolgen, unter anderem kontinuierlich oder intermittierend als Bolus. Inwiefern dies die Therapieeffektivität beeinflusst, wurde in einer randomisierten Analyse mit 80 akut dekompensierten Herzinsuffizienzpatienten untersucht. Es zeigte sich, dass die kontinuierliche Gabe des Diuretikums Furosemid gegenüber einer intermittierenden Bolusapplikation mit einer stärkeren Abnahme der Dekompensationszeichen, welche anhand des Jugularvenendrucks sowie des Vorliegens peripherer Ödeme und einer Orthopnoe beurteilt wurde, assoziiert war [11].

Wenn bei Patienten mit akuter Herzinsuffizienz trotz maximaler diuretischer Therapie keine suffiziente Diurese zu etablieren ist, ist meist der Beginn einer Ultrafiltration notwendig. Entsprechend einer chinesischen Studie mit 100 akut kardial dekompensierten Patienten, kommt dabei der frühzeitigen Nierenersatztherapie eine wesentliche Bedeutung zu [12]. Im randomisierten Vergleich war die frühe Ultrafiltrationsbehandlung innerhalb von 24 h nach Aufnahme gegenüber einer diuretischen Therapie mit Torasemid und Tolvaptan mit einer stärkeren Abnahme des BNP-Wertes und der NYHA-Klasse assoziiert. Es konnten jedoch keine Unterschiede hinsichtlich der Hospitalisierungs- und Mortalitätsraten nach 1 und 3 Monaten nachgewiesen werden [12].

**Figure 3:**
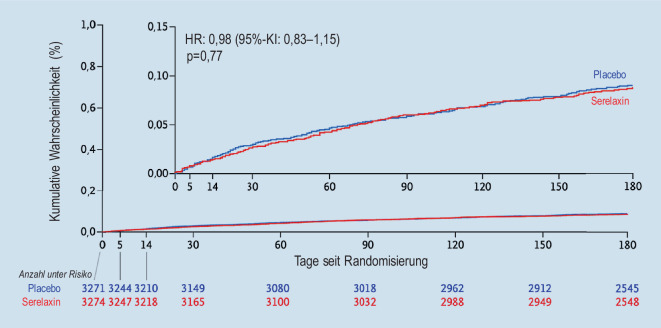

Ein neuartiger Ansatz zur Therapie der akuten Herzinsuffizienz bestand in der Applikation von Serelaxin, einer rekombinanten Form des Schwangerschaftshormons Relaxin. Serelaxin verfügt sowohl über vasodilatorische, antifibrotische als auch über antiinflammatorische Eigenschaften [13]. In der Phase-II-Studie Relax-AHF [14], welche primär die Auswirkungen von Serelaxin auf die Symptomatik von 1161 kardial dekompensierten Patienten untersuchte, zeigte sich eine signifikante Reduktion der Mortalität nach 180 Tagen im Serelaxin-Arm. Die Sterblichkeitsrate nach 180 Tagen in der Relax-AHF-Studie lag jedoch bei unter 1 %, weshalb diese Ergebnisse statistisch nicht aussagekräftig waren. In der nachfolgenden Relax-AHF-II-Studie [15] erfolgte der Einschluss von insgesamt 6545 hospitalisierten Patienten aufgrund einer kardialen Dekompensation. Als primäre Endpunkte dieser randomisierten, placebokontrollierten Studie wurden das Eintreten eines kardiovaskulären Tods nach 180 Tagen und eine klinische Verschlechterung der Herzinsuffizienz nach 5 Tagen, welche eine Intensivierung der Therapie zur Folge hatte, definiert. Hierbei zeigte sich kein signifikanter Vorteil zugunsten einer 48-stündigen intravenöse Therapie mit Serelaxin (Abb. 3).

Ein wichtiger Aspekt, der nicht nur bei der Interpretation der Relax-AHF-II-Studie, sondern generell bei klinischen Studien zur akuten Herzinsuffizienz zu beachten ist, ist der überdurchschnittliche Einschluss von Patienten mit hochnormalen bis hypertensiven Blutdruckwerten. Da die unterschiedlichen Therapieansätze zur akuten Herzinsuffizienz in aller Regel auf dem Prinzip der Entlastung des versagenden Herzens beruhen, sind starke Blutdruckabfälle als unerwünschte Wirkung möglich. Aus Sicherheitsgründen werden daher häufig hohe systolische Schwellenwerte als Voraussetzung für den Studieneinschluss gewählt. In der Relax-AHF-II-Studie beispielsweise musste der systolische Blutdruck bei Studieneinschluss mindestens 125 mm Hg betragen, und der mittlere systolische Ausgangsblutdruck aller 6545 Patienten lag bei 142 mm Hg [15]. In der täglichen klinischen Praxis hingegen weisen Patienten mit akuter kardialer Dekompensation typischerweise niedrignormale bis hypotensive Blutdruckwerte auf. Eine Übertragung der Studienergebnisse auf den klinischen Alltag ist somit nur eingeschränkt möglich. Darüber hinaus ist auch im Hinblick auf zukünftige Studien zu diskutieren, ob eine schnelle Rekompensation über 48 h mit einem nachhaltigen, prognoseverbessernden Effekt über 6 Monate einhergehen kann. Das Prinzip der Relaxinrezeptoraktivierung hingegen wird weiterverfolgt. Aktuell wird ein subkutan zu applizierendes Seralaxinderivat entwickelt, welches durch die kontinuierliche Applikation die Prognose chronisch herzinsuffizienter Patienten verbessern soll.

Ein Eisenmangel zählt zu den wesentlichen Komorbiditäten herzinsuffizienter Patienten und ist mit einer eingeschränkten Belastbarkeit und reduzierten Lebensqualität assoziiert. Die AFFIRM-AHF-Studie [16] untersuchte daher die Therapieeffekte einer intravenösen Eisensubstitution bei insgesamt 1132 Patienten mit akuter Herzinsuffizienz, einer LVEF von weniger als 50 % und einem relevanten Eisenmangel (Ferritinwert < 100 μg/l oder Ferritinwert zwischen 100 und 299 μg/l und Transferrinsättigung < 20 %). Zwar führte die intravenöse Eisensubstitution mit Eisencarboxymaltose im Vergleich zu Placebo zu einer relativen Risikoreduktion des primären Endpunkts (Herzinsuffizenzhospitalisierungen und kardiovaskulärer Tod) um 21 %, jedoch war dieser Effekt statistisch nicht signifikant (*p* = 0,059; [16]). Die Auswertung der sekundären Endpunkte ergab eine stark ausgeprägte Reduktion der Hospitalisierungsraten, wohingegen keine signifikante Reduktion der kardiovaskulären Mortalität nachzuweisen war. Es muss aber beachtet werden, dass sowohl die Rekrutierung als auch das Follow-up der AFFIRM-AHF-Studie zu wesentlichem Anteil während der COVID-19(„coronavirus disease 2019“)-Pandemie erfolgte. Daher ist anzunehmen, dass die Pandemie z. B. aufgrund fehlender Vorstellungen zu Kontrollterminen einen wesentlichen Einfluss auf die Studienergebnisse hatte. Passend dazu, belegte eine Prä-COVID-19-Analyse, in der nur die Studienphase vor Pandemiebeginn berücksichtigt wurde, eine signifikante Reduktion des primären Endpunkts [16].

## Therapie der chronischen Herzinsuffizienz – Wann beginnen nach Dekompensation?

Nach stationärer Behandlung einer kardialen Dekompensation versterben in den ersten 90 Tagen nach Entlassung etwa 10 % der Patienten [17]. Um das Risiko eines frühen Versterbens zu minimieren, ist das frühzeitige Etablieren einer medikamentösen Herzinsuffizienztherapie von großer Bedeutung. So konnte in einer Propensity-Score-gematchten Analyse des GREAT-Registers mit insgesamt 19.980 akut kardial dekompensierten Patienten gezeigt werden, dass sich durch den Beginn einer Therapie mit Betablockern wie auch mit ACE(„angiotensin-converting enzyme“)-Inhibitoren (ACEi) bereits während der Hospitalisierung eine Reduktion der 90-Tages-Sterblichkeit und der 1‑Jahres-Mortalität erreichen lässt [18].

**Figure 4:**
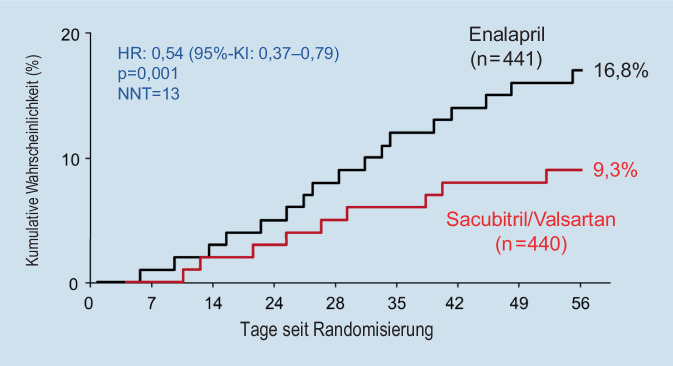

Unklar war jedoch, ob der frühzeitige Beginn einer Therapie mit Sacubitril/Valsartan ebenfalls mit Vorteilen für die Patienten assoziiert ist oder ob sich dies sogar nachteilig auswirkt, z. B. durch eine zu starke Blutdrucksenkung in einer noch instabilen klinischen Situation. Daher wurde die PIONEER-HF-Studie [19] konzipiert, in die insgesamt 881 Patienten eingeschlossen wurden, die aufgrund einer kardialen Dekompensation bei einer Herzinsuffizienz mit reduzierter Ejektionsfraktion („heart failure with reduced ejection fraction“; HFrEF) stationär behandelt wurden. Nach hämodynamischer Stabilisierung wurden die Patienten randomisiert und erhielten entweder eine Therapie mit Sacubitril/Valsartan oder eine Therapie mit Enalapril. Hierbei konnte durch die Therapie mit Sacubitril/Valsartan eine stärkere Abnahme der NT-proBNP(N-terminales Propeptid BNP)-Spiegel (primärer Studienendpunkt) erreicht werden. Gleichzeitig sank das Risiko des kombinierten, sekundären Endpunkts, bestehend aus Tod, Rehospitalisierung bei Herzinsuffizienz, Implantation eines linksventrikulären Assist-Systems und Listung zur Herztransplantation, unter der Therapie mit Sacubitril/Valsartan signifikant um 46 % ([19]; Abb. 4). Diese Effekte waren mit einer relativ niedrigen NNT („number needed to treat“) von 13 assoziiert.

Darüber hinaus bestand nur bei 47 % der eingeschlossenen Patienten eine ACEi-Therapie. Somit konnte die PIONEER-HF-Studie erstmals zeigen, dass eine Therapie mit Sacubitril/Valsartan bei ACEi-naiven Patienten ähnlich wirksam und sicher ist wie bei Patienten mit bereits etablierter ACEi-Therapie. Daher sollte, entsprechend einer Expertenmeinung, der sofortige Beginn mit Sacubitril/Valsartan bei HFrEF-Patienten, die bislang noch keine Behandlung mit einem ACEi erhalten haben, in Erwägung gezogen werden [20].

## Therapie der Herzinsuffizienz bei erhaltener linksventrikulärer Ejektionsfraktion

Die PARADIGM-HF-Studie [21] belegte einen signifikanten Überlebensvorteil durch die Therapie mit Sacubitril/Valsartan bei Patienten mit HFrEF. Ungefähr die Hälfte aller Herzinsuffizienzpatienten leidet aber nicht an einer HFrEF, sondern an einer Herzinsuffizienz mit erhaltener Pumpfunktion („heart failure with preserved ejection fraction“, HFpEF; [22]). Für Patienten mit HFpEF existieren bislang keine Behandlungsmöglichkeiten, für die ein prognostischer Nutzen nachgewiesen werden konnte. Mit Sacubitril/Valsartan erhoffte man sich jedoch eine erste wirksame Therapieoption. Unter anderem kann durch die Therapie mit Sacubitril/Valsartan in die NO(„nitrogen monoxide“)-cGMP(„cyclic guanosine monophosphate“)-PKG(„protein kinase G“)-Achse eingriffen werden, welche eine entscheidende Rolle bei der Ausbildung einer diastolischen Dysfunktion spielt [23, 24]. Darüber hinaus geht die Neprilysininhibition mit erhöhten Spiegeln natriuretischer Peptide einher, die eine Vasodilatation und verstärkte Diurese bewirken und somit das Herz entlasten [25].

Die Therapie mit Sacubitril/Valsartan wurde daher in der PARAGON-HF-Studie [26] mit 4822 eingeschlossenen HFpEF-Patienten (LVEF ≥ 45 %) untersucht. Hierbei wurde die signifikante Reduktion des primären Studienendpunkts „kardiovaskulärer Tod und alle Herzinsuffizienzhospitalisierungen“ verfehlt.

Eine entscheidende Frage nach Veröffentlichung der PARAGON-HF-Studie [26] war, ob die gesteigerte Konzentration und Aktivität von Neprilysin überhaupt einen spezifischen Pathomechanismus bei Patienten mit HFpEF darstellen. Dagegen sprechen Daten einer retrospektiven Analyse mit 144 HFpEF-Patienten, bei der erhöhte Neprilysinspiegel nicht mit einer erhöhten Rate an Herzinsuffizienzhospitalisierungen oder Tod vergesellschaftet waren [27]. Bei Patienten mit HFrEF hingegen korrelieren erhöhte Neprilysinkonzentrationen mit einer ungünstigen Prognose. An 1069 HFrEF-Patienten konnte gezeigt werden, dass das Risiko des kombinierten Endpunkts aus Herzinsuffizienzhospitalisierung und kardiovaskulärem Tod um 20 % erhöht war, falls der Neprilysinserumspiegel oberhalb des Medians lag [28]. Die pathophysiologische Grundlage der Neprilysinhibition bei Patienten mit HFpEF ist dementsprechend fraglich.

Trotzdem ließ sich in der PARAGON-HF-Studie [26] eine zwar nicht signifikante, aber nominelle Risikoreduktion von 13 % unter der Sacubitril/Valsartan-Therapie beobachten. Dies wurde zunächst auf eine effizientere Blutdruckreduktion unter Sacubitril/Valsartan zurückgeführt, welche im Vergleich zu einer Valsartantherapie etwa doppelt so stark ausgeprägt ist [29]. Eine Post-hoc-Analyse der PARAGON-HF-Studie zeigte jedoch, dass die erwähnte nominelle Reduktion des primären Endpunkts nicht durch die stärkere Abnahme des systolischen Blutdrucks unter einer Therapie mit Sacubitril/Valsartan zustande kam [30].

Des Weiteren wird zunehmend darüber diskutiert, ob es sinnvoll und ausreichend ist, die Einteilung der Herzinsuffizienzformen und damit auch die Definition der HFpEF einzig anhand der LVEF vorzunehmen [31]. Dadurch sind nämlich signifikante Überschneidungen der Phänotypen zwischen den LVEF-basierten Subgruppen möglich [32, 33]. Aus diesen Gründen hat die HFA der ESC ein Konsensusdokument veröffentlicht, in dem die Autoren die Anwendung eines umfassenden diagnostischen Algorithmus zur Abklärung eines HFpEF-Verdachts empfehlen [31]. Hierbei basiert die Diagnose der HFpEF nicht mehr ausschließlich auf der LVEF-Evaluation, sondern es werden auch andere Aspekte, wie z. B. klinische Charakteristika, EKG-Befunde, echokardiographische Parameter einer diastolischen Dysfunktion, Laborparameter sowie hämodynamische Parameter, berücksichtigt (Tab. 1). Darüber hinaus sollte bei allen HFpEF-Patienten eine ätiologische Abklärung angestrebt werden, um spezifische Ursachen (z. B. M. Fabry oder kardiale Amyloidose) nachweisen zu können. Dies gilt in gleicher Weise auch für Patienten mit HFrEF. In der PARAGON-Studie [26] hingegen beruhten die Definition der HFpEF und damit die Kriterien zum Studieneinschluss primär auf der erhobenen LVEF, die mindestens 45 % betragen musste. Eine ätiologische Abklärung wurde ebenfalls nicht durchgeführt. Somit könnten die Therapieeffekte und damit auch die Studienergebnisse signifikant beeinflusst worden sein. Die Heterogenität des HFpEF-Syndroms wird ferner durch die Ergebnisse der Subgruppenanalysen der PARAGON-HF-Studie unterstrichen. So profitierten beispielsweise Frauen von der Therapie mit Sacubitril/Valsartan insbesondere durch eine Reduktion der Herzinsuffizienzhospitalisierungen, während dies bei Männern nicht der Fall war [34]. Zudem nahm das Risiko des primären Endpunkts bei Patienten mit einer LVEF zwischen 45 und 57 % um 22 % ab, wohingegen bei Patienten mit einer LVEF oberhalb des Medians (57 %) kein Therapieeffekt nachzuweisen war [26]. In einer Post-hoc-Analyse, die die Wirkung von Sacubitril/Valsartan bei allen Patienten der PARADIGM-HF- und PARAGON-HF-Studien in Abhängigkeit der LVEF untersuchte, war die Gabe von Sacubitril/Valsartan bis zu einer LVEF von 55 % mit einem signifikanten Überlebensvorteil assoziiert [35]. Zu vergleichbaren Ergebnissen kamen auch Post-hoc-Analysen anderer, großer HFpEF-Studien, wie der CHARM-Preserved- [36] oder der TOPCAT-Studie [37], die einen Nutzen für Patienten mit einer LVEF unter 55 % nahelegen [38]. Für eine Betablockertherapie konnten positive Therapieeffekte bis zu einer LVEF von weniger als 50 % nachgewiesen werden [39]. Es ist jedoch anzumerken, dass der LVEF-Bereich zwischen 45 und 55 %, entsprechend der ESC-Definition, nicht mehr ausschließlich Patienten mit einer HFpEF betrifft, sondern auch solche, die an einer Herzinsuffizienz mit mittelgradig eingeschränkter Ejektionsfraktion („heart failure with mid-range reduced ejection fraction“, HFmEF) leiden [1].

**Table 1:** 

***P***	Prätestwahrscheinlichkeit	Symptome	Orthopnoe, Belastungsdyspnoe (NYHA II–III), eingeschränkte Belastbarkeit
		Komorbiditäten, Risikofaktoren	Arterielle Hypertonie, Diabetes mellitus, Adipositas, Vorhofflimmern, Alter
		EKG	Positive LVH-Indizes, P mitrale, Vorhofflimmern
		Ergometrie, 6‑Minuten-Gehtest, kardiopulmonale Belastungstests	Chronotrope Inkompetenz, maximale ergometrische Belastbarkeit ≤ 75 % des altersüblichen Durchschnittswerts, 6‑Minuten-Gehstrecke ≤ 300m, VO_2_max ≤ 20 ml/kg/min und „VE/VCO_2_ slope“ ≥ 30
**E**	Echokardiographie/natriuretische-Peptide-Score (HFA-PEFF-Score)	Morphologische Parameter	Majorkriterien: LAVI > 34 ml/m^2^ **oder **LVMI ≥ 149/122 g/m^2^ (m/w) und RWT > 0,42 Minorkriterien: LAVI 29–34 ml/m^2^ **oder **LVMI > 115/95 g/m^2^ (m/w) **oder **LV-Wanddiameter ≥ 12 mm
		Funktionelle Parameter	Majorkriterien: septales e’ < 7 cm/s **oder** laterales e’ < 10 cm/s **oder **Durchschnitts-E/e’ ≥ 15 **oder **Trikuspidalinsuffizienzgeschwindigkeit > 2,8m/s (PASP > 35 mm Hg) Minorkriterien: Durchschnitts-E/e’ 9–14 **oder** GLS < 16 %
		Natriuretische Peptide bei Sinusrhythmus	Majorkriterien: NT-proBNP > 220 pg/ml **oder **BNP > 80 pg/ml Minorkriterien: NT-proBNP = 125–220 pg/ml **oder** BNP = 35–80 pg/ml
		Natriuretische Peptide bei Vorhofflimmern	Majorkriterien: NT-proBNP > 660 pg/ml **oder** BNP > 240 pg/ml Minorkriterien: NT-proBNP = 365–660 pg/ml **oder** BNP= 105–240 pg/ml
**F1**	Funktionelle Tests bei unklaren Fällen	Stressechokardiographie	Anstieg des Durchschnitts E/e’ ≥ 15 unter Belastung mit **oder **ohne Anstieg der Trikuspidalinsuffizienzgeschwindigkeit > 3,4 m/s unter Belastung
		Invasive Messung der Hämodynamik	Linksherzkatheter: LVEDP ≥ 16 mm Hg in Ruhe, eingeschränkte LV-Relaxation (tau, τ > 48 ms) in Ruhe Rechtsherzkatheter: mPCWP ≥ 15 mm Hg in Ruhe, Anstieg des PCWP ≥ 25 mm Hg unter Belastung
**F2**	Finale ätiologische Abklärung	CMR/Szintigraphie/CT/PET	Hypertrophe Kardiomyopathie, kardiale Amyloidose, Malignome
		Spezielle Labordiagnostik, genetische Analysen	Morbus Wilson, Morbus Fabry, hypertrophe Kardiomyopathie, …
		Biopsieentnahme	Myokardial und nichtmyokardial

*NYHA* New York Heart Association, *BNP* „brain natriuretic peptide“, *CMR* kardiale Magnetresonanztomographie, *GLS* globaler Longitudinal-Strain, *LAVI* linksatrialer Volumenindex, *LVEDP* linksventrikulärer enddiastolischer Druck, *LVH* linksventrikuläre Hypertrophie, *LVMI* linksventrikulärer Massenindex, *NT-proBNP* N-terminales Propeptid BNP, *PASP* systolischer pulmonalarterieller Druck, *PCWP* pulmonalkapillärer Wedge-Druck, *RWT* relative Wanddicke, *VCO*_*2*_ Kohlendioxidaufnahme, *VE* Ventilation, *VO*_*2*_*max* maximale Sauerstoffaufnahme, *CT* Computertomographie, *PET* Positronenemissionstomographie, *mPCWP* mittlerer pulmonalkapillärer Wedge-Druck

Bereits vor Veröffentlichung der PARAGON-HF-Studie wurde zudem die PARALLAX-Studie [40] initiiert, welche die Auswirkungen einer Therapie mit Sacubitril/Valsartan insbesondere auf funktionelle Parameter untersuchen sollte. Innerhalb der PARALLAX-Studie wurden Patienten mit einer LVEF von mehr als 40 % eingeschlossen. In der Kontrollgruppe erhielten die Patienten in Abhängigkeit von ihrer Medikation bei Studieneinschluss entweder eine Valsartantherapie (Angiotensin-II-Rezeptor-Blocker [ARB] bei Studieneinschluss), eine Enalapriltherapie (ACEi bei Studieneinschluss) oder eine Placebotherapie (weder ARB noch ACEi bei Studieneinschluss). Die Studie ist bislang noch nicht publiziert, die Ergebnisse wurden aber kürzlich beim digitalen ESC-Kongress vorgestellt. Durch die Behandlung mit Sacubtril/Valsartan konnte im Gegensatz zur Kontrollgruppe eine Abnahme der humoralen NT-proBNP-Konzentration um 16 % erzielt werden (*p* < 0,001; [40]). Die Gehstrecke im 6‑Minuten-Gehtest zeigte sich jedoch unverändert. Des Weiteren hatte die Therapie mit Sacubitril/Valsartan keine Auswirkungen auf das NYHA-Stadium und die Lebensqualität der Patienten, welche mit Hilfe des Kansas City Cardiomyopathy Questionnaire (KCCQ) ermittelt wurde. In einer zusätzlich vorgestellten Post-hoc-Analyse war die Behandlung mit Sacubitril/Valsartan zwar mit einer geringeren Rate an kardiovaskulären Todesfällen sowie Herzinsuffizienzhospitalisierungen assoziiert, jedoch handelt es sich dabei um eine nichtadjustierte Analyse [40].

Zusammenfassend konnte für die Therapie mit Sacubitril/Valsartan kein Überlebensvorteil bei Patienten mit HFpEF nachgewiesen werden. Möglicherweise können aber Patienten mit HFmEF von einer Behandlung mit Sacubitril/Valsartan sowie von einer Therapie mit Betablockern, ACEi/ARB und Spironolacton profitieren. Ein wichtiger Aspekt für zukünftige Studien ist zudem die genaue und einheitliche Definition des HFpEF-Syndroms, welche neben der LVEF auch andere diagnostische Kriterien, wie den HFA-PEFF(P: „pretest assessment“, E: „diagnostic workup with echocardiogram and natriuretic peptide score“, F: „advanced workup with functional testing in case of uncertainty“, F: „final etiological workup“)-Score, berücksichtigen sollte [41]. In diesem Kontext kommt der eindeutigen Klärung der Erkrankungsursache eine wesentliche Bedeutung zu, um spezifische Ursachen der HFpEF zu identifizieren und gezielt zu behandeln. Hierzu zählt z. B. der Beginn einer Tafamidistherapie bei Patienten mit Transthyretinamyloidose (ATTR).

## Therapie mit SGLT2-Inhibitoren bei chronischer Herzinsuffizienz und chronischer Niereninsuffizienz

Vertreter der Gliflozine, wie Empagliflozin oder Dapagliflozin, inhibieren den Natrium-Glukose-Kotransporter 2 („sodium-glucose linked transporter 2“, SGLT2) der proximalen Nierentubuli, wodurch die Glukoserückresorption aus dem Primärharn reduziert und letztlich eine renale Glukosurie hervorgerufen wird. Somit kann eine Abnahme der Blutglukosekonzentration erreicht werden. Daher fanden die SGLT2-Inhibitoren zunächst im Rahmen der Diabetesbehandlung Anwendung. In der EMPAREG-OUTCOME-Studie konnte neben einer signifikanten Abnahme kardiovaskulärer Endpunkte insbesondere auch eine signifikante Reduktion herzinsuffizienzbedingter Hospitalisierungen bei Diabetikern mit einem erhöhten kardiovaskulären Risikoprofil beobachtet werden [42]. Die zugrunde liegenden Mechanismen sind weiterhin nicht eindeutig geklärt und Gegenstand kontroverser Diskussionen. Hierzu zählen eine Verbesserung der kardialen Energetik durch eine vermehrte Ketonkörperproduktion sowie eine Abnahme der Vor- und Nachlast [43, 44]. Darüber hinaus war lange fraglich, ob diese positiven Effekte ausschließlich bei Patienten mit Diabetes eintreten oder ob auch Herzinsuffizienzpatienten ohne Diabetes von einer SGLT2-Therapie profitieren können.

**Figure 5:**
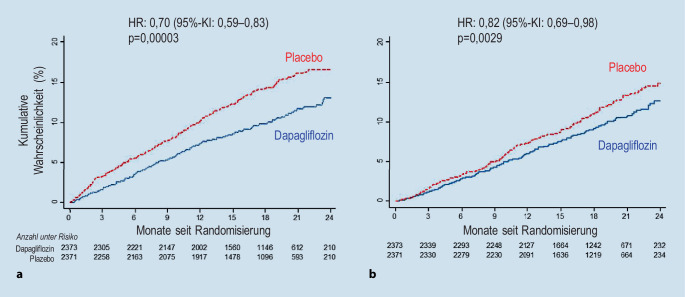

Aus diesem Grund wurde die DAPA-HF-Studie [45] durchgeführt, in der eine Therapie mit Dapagliflozin gegenüber einer optimalen, leitliniengerechten Herzinsuffizienztherapie randomisiert verglichen wurde. Insgesamt wurden 4744 Patienten mit einer HFrEF (LVEF ≤ 40 %) im NYHA-Stadium II–IV unabhängig vom Vorliegen eines Diabetes eingeschlossen und über einen medianen Zeitraum von 18,2 Monaten nachverfolgt. Der primäre kombinierte Endpunkt setzte sich aus den Komponenten Notwendigkeit einer intravenösen diuretischen Therapie, Herzinsuffizienzhospitalisierung und kardiovaskulärer Tod zusammen. Die DAPA-HF-Studie belegte eine signifikante Abnahme des primären Endpunkts um 26 % durch die Therapie mit Dapagliflozin, wobei sich die NNT auf 21 belief [45]. Des Weiteren wurde bei den dapagliflozinbehandelten Patienten eine Abnahme der Herzinsuffizienzhospitalisierungsrate um 30 %, der kardiovaskulären Sterblichkeit um 18 % und der Gesamtmortalität um 17 % dokumentiert (Abb. 5). In einer präspezifizierten Analyse konnte zudem gezeigt werden, dass die relative Risikoreduktion bei Diabetikern und Nichtdiabetikern ähnlich ausgeprägt war, wobei Diabetiker einem höheren absoluten Risiko ausgesetzt waren. Außerdem profitierten unabhängig vom Hämoglobin‑A_1c_(HbA_1c_)-Wert alle Patienten signifikant von einer Therapie mit Dapagliflozin [46]. Darüber hinaus war der Therapieeffekt unabhängig vom Alter der Patienten nachzuweisen [47]. Ferner ging die Therapie mit Dapagliflozin mit einer signifikanten Verbesserung der Symptomatik, der körperlichen Belastbarkeit und der Lebensqualität, welche mit Hilfe des KCCQ objektiv gemessen wurden, einher [48]. Ein wichtiger Aspekt ist auch, dass Dapagliflozin bei Patienten mit HFrEF nur zu einer geringen Reduktion des systolischen Blutdrucks führt, was einen entscheidenden Vorteil gegenüber einer neuroendokrinen Therapie darstellt [49]. Des Weiteren konnten durch die Therapie mit Dapagliflozin unabhängig vom systolischen Blutdruck sowie von der Diuretikadosis bei Einschluss in die DAPA-HF-Studie signifikante Effekte auf die Prognose der Patienten erzielt werden [49, 50].

**Figure 6:**
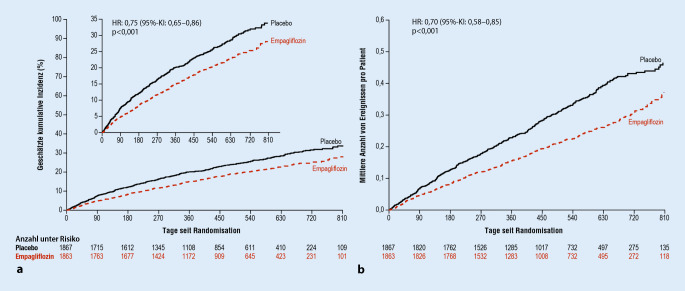

Während des ESC-Kongresses 2020 wurde nun außerdem die EMPEROR-Reduced-Studie vorgestellt, die die Wirksamkeit einer Therapie mit Empagliflozin gegenüber einer leitliniengerechten Herzinsuffizienztherapie bei 3730 Herzinsuffizienzpatienten mit einer LVEF von 40 % oder weniger verglich [51]. Durch die zusätzliche Therapie mit Empagliflozin wurde das Risiko des primären Endpunkts (kardiovaskuläre Sterblichkeit und Herzinsuffizienzhospitalisierung) signifikant reduziert (Abb. 6). Analog zur DAPA-HF-Studie ließ sich in der Subgruppenanalyse eine vergleichbare Risikoabnahme bei Patienten sowohl mit als auch ohne Diabetes nachweisen [51]. Darüber hinaus war die Therapie mit Empagliflozin mit einem geringeren Risiko eines kombinierten „renalen Endpunkts“ assoziiert, welcher sich aus der Notwendigkeit einer chronischen Dialysetherapie, einer Nierentransplantation sowie einer relevanten und anhaltenden Abnahme der geschätzten glomerulären Filtrationsrate („estimated GFR“, eGFR) zusammensetzte [51].

Die Effekte einer SGLT2-Inhibition auf die Nierenfunktion wurden ferner in der randomisierten DAPA-CKD-Studie [52] untersucht. In die Studie wurden 4304 Patienten mit einer chronischen Niereninsuffizienz eingeschlossen, die eine eGFR zwischen 25 und 75 ml/min/1,73 m^2^ und einen Urin-Albumin/Kreatinin-Quotienten zwischen 200 und 5000 mg/g aufwiesen. Während die Patienten in der Kontrollgruppe eine Standardtherapie mit einem ACEi oder einem ARB erhielten, erfolgte in der Kontrollgruppe eine zusätzliche Therapie mit Dapagliflozin. Innerhalb eines medianen Nachbeobachtungszeitraums von 2,4 Jahren konnte das relative Risiko des primären Studienendpunkts (kombinierter Endpunkt aus ≥ 50 % anhaltende Abnahme der eGFR, einsetzendem Nierenversagen oder Tod aufgrund kardiovaskulärer oder renaler Ursachen) mittels Dapagliflozin um 39 % reduziert werden [52].

Wie eine Metaanalyse [53] zeigen konnte, sind die beiden Substanzen Empagliflozin und Dapagliflozin in ihrer Wirkung bei Patienten mit HFrEF vergleichbar. Hierzu erfolgte die Evaluation einer SGLT2-Therapie bei allen Patienten, die in die DAPA-HF- oder in die EMPEROR-Reduced-Studie eingeschlossen wurden. Es zeigte sich, dass durch eine SGLT2-Inhibitor-Therapie eine Abnahme der Gesamtmortalität, der kardiovaskulären Mortalität, der Herzinsuffizienzhospitalisierungen und des kombinierten renalen Endpunkts (≥ 50 % anhaltende Abnahme der eGFR, terminale Niereninsuffizienz oder Tod aufgrund renaler Ursachen) erreicht wird [53]. Darüber hinaus ließen sich die positiven Therapieeffekte unabhängig von Alter, Geschlecht, Vorliegen einer Diabeteserkrankung, eGFR bei Studieneinschluss und Vorbehandlung mit Sacubtril/Valsartan nachweisen.

Aktuell werden weitere Untersuchungen zur SGLT2-Inhibition bei Patienten mit Herzinsuffizienz durchgeführt. Durch die EMPEROR-Preserved-Studie mit Empagliflozin und durch die DELIVER-Studie mit Dapagliflozin soll geklärt werden, ob die SGLT2-Therapie eine Abnahme kardiovaskulärer Endpunkte bei Patienten mit HFpEF bewirken kann. Die Randomisationsphase der EMPEROR-Preserved-Studie wurde bereits Anfang 2020 abgeschlossen. Das DETERMINE-Studienprogramm hingegen konzentriert sich primär auf die Änderung der körperlichen Belastbarkeit unter einer Therapie mit Dapagliflozin bei HFrEF- (DETERMINE-Reduced) und HFpEF-Patienten (DETERMINE-Preserved). Im EMPERIAL-Studienprogramm konnte aber bereits gezeigt werden, dass eine Therapie mit Empagliflozin sowohl bei Patienten mit HFpEF (EMPERIAL-Preserved) als auch bei Patienten mit HFrEF (EMPERIAL-Reduced) keinen signifikanten Einfluss auf die körperliche Belastbarkeit hat, welche anhand der Gehstrecke im 6‑Minuten-Gehtest ermittelt wurde [54].

## Behandlung mit Guanylatzyklasemodulatoren bei chronischer Herzinsuffizienz

**Figure 7:**
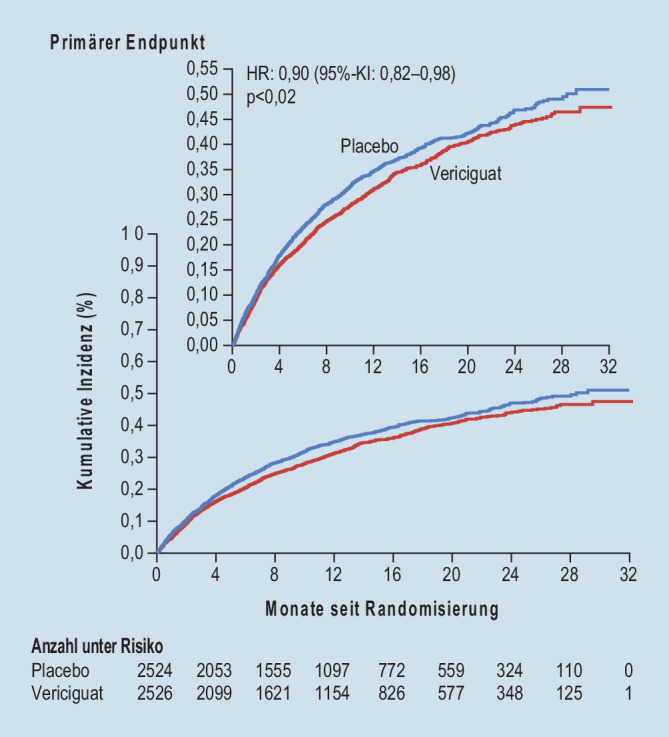

Die Guanylatzyklase stimuliert die Bildung des Second Messengers cGMP („cyclic guanosine monophosphate“), welcher unter anderem eine verstärkte Vasorelaxation, eine verminderte Proliferation glatter Muskelzellen sowie eine Abnahme der Leukozytenrekrutierung und der Thrombozytenaggregation bewirkt [55]. Bei Patienten mit chronischer Herzinsuffizienz kann jedoch eine Oxidation der Guanylatzyklase einsetzen, wodurch diese nicht mehr endogen aktiviert werden kann und es zu einer Abnahme der cGMP-Spiegel kommt [56]. Mithilfe löslicher Guanylatzyklaseaktivatoren bzw. -stimulatoren wird dieser Pathomechanismus gezielt adressiert. Die randomisierte, placebokontrollierte VICTORIA-Studie [57] untersuchte dieses Therapiekonzept und schloss hierzu 5050 Patienten mit HFrEF (LVEF < 45 %) nach einer kardialen Dekompensation ein. Durch die Behandlung mit Vericiguat konnte eine signifikante Reduktion des kombinierten primären Endpunkts, bestehend aus erstmaliger Herzinsuffizienzhospitalisierung oder kardiovaskulärem Tod, erreicht werden (Abb. 7; [57]). Die relative Risikoreduktion betrug dabei 10 %. Vergleichbare Endpunkte wurden in der PARADIGM-HF- [21] und der DAPA-HF-Studie [45] untersucht. In der PARADIGM-HF-Studie konnte das relative Risiko durch die Therapie mit Sacubitril/Valsartan um 20 % gesenkt werden [21]. In der DAPA-HF-Studie ließ sich durch die Behandlung mit Dapagliflozin eine Abnahme des relativen Risikos um 26 % nachweisen [45]. Somit sind die positiven Effekte einer Therapie mit Vericiguat auf den ersten Blick eher gering ausgeprägt. Es muss jedoch beachtet werden, dass in der VICTORIA-Studie 41 % der eingeschlossenen Patienten Symptome einer fortgeschrittenen Herzinsuffizienz, entsprechend einer NYHA-Klasse III oder IV, aufwiesen [57]. Der mediane NT-proBNP-Wert war mit 2816 pg/ml ebenfalls deutlich erhöht. In PARADIGM-HF [21] und DAPA-HF [45] hingegen befanden sich nur 25 % bzw. 32 % der eingeschlossenen Patienten im NYHA-Stadium III oder IV, und der mediane NT-proBNP-Wert lag bei 1608 pg/ml bzw. 1437 pg/ml. Passend dazu zeichneten sich die Patienten in VICTORIA [57] durch ein grundsätzlich hohes absolutes Risiko aus. Trotz der geringer ausgeprägten relativen Risikoreduktion konnte daher eine absolute Risikoreduktion von 4,2 % erreicht werden, welche mit der absoluten Risikoreduktion in der PARADIGM-HF- (4,7 %; [21]) und in der DAPA-HF-Studie (4 %; [45]) vergleichbar ist.

## Telemedizin bei Herzinsuffizienz

Die Überwachung des Flüssigkeitshaushalts stellt bei Patienten mit chronischer Herzinsuffizienz eine essenzielle Maßnahme dar, um drohende kardiale Dekompensationen frühzeitig zu detektieren und somit Herzinsuffizienzhospitalisierungen zu vermeiden. Das Telemonitoring des pulmonalarteriellen Drucks mit Hilfe des CardioMEMS-Sensors konnte sich hierbei als effektive Therapiemaßnahme beweisen. Nach den überzeugenden Resultaten der US-amerikanischen CHAMPION-Studie [58] erfolgte die Evaluation der Methode innerhalb Europas mittels der MEMS-HF-Studie. Hierbei handelte es sich um eine prospektive, jedoch nicht placebokontrollierte Studie, in die 234 symptomatische Herzinsuffizienzpatienten eingeschlossen wurden [59]. Der Studieneinschluss war unabhängig von der LVEF, sodass Patienten sowohl mit HFrEF (LVEF < 40 %) als auch mit HFmEF und HFpEF (LVEF ≥ 40 %) berücksichtigt wurden. Es zeigte sich, dass die Hospitalisierungsraten aufgrund einer Herzinsuffizienz nach Implantation des CardioMEMS-Devices um 62 % reduziert werden konnten [60]. Darüber hinaus ließ sich eine Besserung der Lebensqualität nach Beginn der CardioMEMS-gesteuerten Therapie nachweisen, welche unter anderem anhand des KCCQ objektiv gemessen wurde. Komplikationen durch die CardioMEMS-Implantation sowie ein Versagen des Sensors wurden lediglich bei 4 Patienten (1,7 %) dokumentiert und führten nicht zum Tod [60]. Zur weiteren Evaluation des CardioMEMS-Telemonitorings wurde bereits die randomisierte, placebokontrollierte GUIDE-HF-Studie (NCT03387813) initiiert, welche rund 3600 Patienten mit symptomatischer Herzinsuffizienz einschließen soll. Die Anwendung telemedizinischer Verfahren spielt auch eine entscheidende Rolle zur Reduktion von Rehospitalisierungen nach stationärer Behandlung einer akuten Herzinsuffizienz [61].

## Neue Ansatzpunkte zur Behandlung der Herzinsuffizienz

### Klonale Hämatopoese

Durch Genmutationen während der Zellteilung myeloischer Stammzellen können sog. prämaligne Stammzellen entstehen, die klonal expandiert sind und letztlich mutierte myeloische Zellen in den Blutkreislauf abgeben [62]. Dabei steigt die Wahrscheinlichkeit einer Genmutation mit zunehmendem Alter an, sodass im Alter von 70 Jahren bei über 10 % aller Patienten mutierte Zellen nachzuweisen sind [63]. Obwohl eine solche klonale Hämatopoese mit einer erhöhten Inzidenzrate hämatologischer Erkrankungen assoziiert ist, bildet sich nur bei wenigen Patienten eine tatsächliche Malignität, wie z. B. eine akute myeloische Leukämie, aus. Daher wurde der Begriff der klonalen Hämatopoese von unbestimmtem Potenzial („clonal hematopoiesis of indeterminate potential“, CHIP) eingeführt, der anzuwenden ist, wenn bei mehr als 2 % aller peripheren Leukozyten Mutationen vorliegen [63]. Insbesondere die CHIP-Mutationen der Gene *DNMT3A* und *TET2*, welche zu den 4 häufigsten zählen, führen zu einer gesteigerten Biosynthese proinflammatorischer Zytokine und werden daher als ein pathophysiologischer Mechanismus der Atherogenese diskutiert. So ging in einer Studie an 200 Patienten mit ischämischer Kardiomyopathie der Nachweis einer CHIP-Mutation, insbesondere der Gene *DNMT3A* und *TET2*, mit einer erhöhten Sterblichkeit bzw. mit einem höheren Risiko einer Herzinsuffizienzhospitalisierung einher [64]. Zusätzlich zeigte sich, dass ab einem Alter von 50 Jahren die Wahrscheinlichkeit einer CHIP-Mutation stetig zunimmt. Möglicherweise könnte das Vorbeugen einer CHIP-Mutation einen neuartigen Therapieansatz für die Zukunft darstellen.

### Omecamtiv-Mecarbil

Klassische, positiv-inotrope Substanzen können bei akuter Herzinsuffizienz über das Einwirken in die Kalziumhomöostase der Myozyten zwar zu einer kurzfristigen Stabilisierung der Patienten beitragen, sind aber langfristig mit einer erhöhten Mortalitätsrate assoziiert [65]. Neben diesen Kalzitropika (Dobutamin, Dopamin, Katecholamine, PDE[Phosphodiesterase]-5-Inhibitoren) stehen aber noch weitere inotrope Substanzen zur Verfügung, die entsprechend ihren Angriffspunkten zur Gruppe der Mitotropika (Mitochondrien als Angriffspunkt) oder zur Gruppe der Myotropika (Myosin-Aktin-Interaktion als Angriffspunkt) gezählt werden [66]. Ein wichtiger Vertreter der Myotropika stellt Omecamtiv-Mecarbil dar. Über eine direkte Myosinaktivierung führt Omecamtiv-Mecarbil zu einer energieunabhängigen Zunahme der Kontraktilität, während die Kontraktionszeit und somit die Dauer der Systole verlängert werden [67]. Obwohl dieser Effekt einer Frequenzabhängigkeit unterliegt, ist er über das gesamte Frequenzspektrum zu beobachten. Bereits aus Untersuchungen in den 1960er-Jahren geht hervor, dass die Herzinsuffizienz mit einer signifikanten Abnahme der systolischen Ejektionszeit vergesellschaftet ist und es sich hierbei um einen pathophysiologischen Mechanismus handeln könnte [68]. Somit unterscheidet sich Omecamtiv-Mecarbil wesentlich von der Wirkung der Kalzitropika, die eine zusätzliche Reduktion der Kontraktionsdauer begünstigen. In der COSMIC-HF-Studie [69] konnte bereits gezeigt werden, dass eine Therapie mit Omecamtiv-Mecarbil bei stabilen Patienten mit chronischer HFrEF zu einer Abnahme der NT-proBNP-Spiegel und der Herzfrequenz sowie zu einer Zunahme der LVEF führt. Die anschließend initiierte GALACTIC-HF-Studie [70] untersuchte die Effekte einer Therapie mit Omecamtiv-Mecarbil auf harte klinische Endpunkte. Im Vergleich zu Placebo reduzierte Omecamtiv-Mecarbil den kombinierten primären Endpunkt (kardiovaskulärer Tod, Herzinsuffizienzhospitalisierung, dringende Behandlung einer Herzinsuffizienz) signifikant [70]. Die relative Risikoreduktion betrug jedoch nur 8 %. Außerdem konnte keine Abnahme der kardiovaskulären Sterblichkeit unter Omecamtiv-Mecarbil erreicht werden [70]. Aus diesen Gründen kündigte einer der Sponsoren an, die weitere Entwicklung des Wirkstoffs nicht zu unterstützen. Eine zukünftige Marktzulassung von Omecamtiv-Mecarbil ist somit durchaus fraglich.

## Mögliche Leitlinienempfehlung in der Zukunft

**Figure 8:**
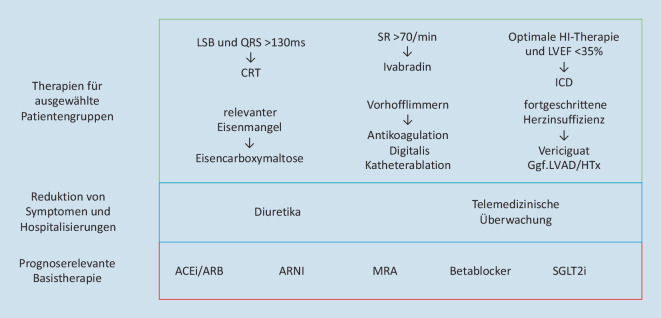

Aktuell werden die ESC-Leitlinien zur akuten und chronischen Herzinsuffizienz überarbeitet. Die aktualisierte Auflage wird vermutlich Ende 2021 bis spätestens Anfang 2022 fertiggestellt sein und anschließend veröffentlicht. Es ist wahrscheinlich, dass das bisherige Konzept der Stufentherapie verlassen wird. Diese Annahme ist beispielsweise auf die Ergebnisse einer großen Metaanalyse mit insgesamt 15.880 HFrEF-Patienten aus der EMPHASIS-HF- [71], der PARADIGM-HF- [21] und der DAPA-HF-Studie [45] zurückzuführen [72]. Hierbei konnte ein signifikanter Vorteil einer maximalen medikamentösen Herzinsuffizienztherapie (Angiotensin-Rezeptor-Neprilysin-Inhibitor [ARNI], Betablocker, Mineralokortikoidrezeptorantagonist [MRA] und SGLT2-Inhibitor) gegenüber einer konventionellen Therapie (ACEi oder ARB und Betablocker) hinsichtlich des kombinierten Endpunkts (erste Herzinsuffizienzhospitalisierung oder kardiovaskulärer Tod) nachgewiesen werden [72]. Bei Beginn einer maximalen Therapie im Alter von 55 Jahren konnte im Vergleich zu einer konventionellen Behandlung eine zusätzliche Lebenserwartung von 6,3 Jahren erreicht werden [72]. Daher ist davon auszugehen, dass in den zukünftigen Leitlinien eine Kombination aus ACEi/ARB bzw. ARNI, Betablocker, MRA und SGLT2-Inhibitor als Basistherapie empfohlen wird (Abb. 8).

## Fazit für die Praxis

- Trotz wesentlicher Fortschritte im Bereich der Herzinsuffizienzdiagnostik und -therapie bleibt die Herzinsuffizienz mit einer hohen Morbidität und Mortalität assoziiert. Eine leitliniengerechte Behandlung herzinsuffizienter Patienten ist daher essenziell.
- Wie die PIONEER-Studie deutlich gemacht hat, ist die frühzeitige Etablierung einer Neprilysininhibition bei Patienten mit HFrEF („heart failure with reduced ejection fraction“) mit Überlebensvorteilen assoziiert und sollte auch bei ACE(„angiotensin-converting enzyme“)-Inhibitor-naiven Patienten in Betracht gezogen werden.
- Im Gegensatz dazu stehen die ernüchternden Resultate der PARAGON-HF-Studie, sodass weiterhin keine prognostisch wirksamen Therapieoptionen für Patienten mit HFpEF („heart failure with preserved ejection fraction“) zur Verfügung stehen.
- Die Therapie mit SGLT2(„sodium-glucose linked transporter 2“)-Inhibitoren bei Patienten mit HFrEF hat sich in 2 großen randomisierten, placebokontrollierten Studien als prognostisch wirksam erwiesen und wird sich vermutlich als ein wichtiger Bestandteil der zukünftigen medikamentösen Behandlung der HFrEF etablieren.
- Mit Omecamtiv-Mecarbil steht nun erstmals ein Therapieansatz bei der akuten Herzinsuffizienz zur Verfügung, für den ein signifikanter Nutzen im Rahmen der multizentrischen, randomisierten, placebokontrollierten GALACTIC-HF-Studie nachgewiesen werden konnte. Aktuell ist es aber durchaus fraglich, ob Omecamtiv-Mecarbil eine Marktzulassung erhalten wird.
